# Mutational analysis of renal angiomyolipoma associated with tuberous sclerosis complex and the outcome of short-term everolimus therapy

**DOI:** 10.1038/s41598-019-49814-6

**Published:** 2019-10-04

**Authors:** Jianxin Ni, Fengqi Yan, Weijun Qin, Lei Yu, Geng Zhang, Fei Liu, Xiaojian Yang, Bo Yang, Chunlin Hao, Teng Wang, Pengfei Liu, Jianlin Yuan, Guojun Wu

**Affiliations:** 1 Department of Urology, Xi Jing Hospital, the Fourth Military Medical University, Shaanxi, Xian 710032 China; 2 Department of Urology, Tang Du Hospital, the Fourth Military Medical University, Shaanxi, Xian 710038 China

**Keywords:** Kidney diseases, Urological manifestations

## Abstract

To identify clinical characteristics and mutation spectra in Chinese patients with renal angiomyolipoma (AML) associated with the tuberous sclerosis complex (TSC, TSC-AML), examined the efficacy and safety of short-term everolimus therapy (12 weeks). We analyzed the frequency distribution of each TSC-related clinical feature and investigated gene mutations by genetic testing. Some subjects received everolimus for 12 weeks at a dose of 10 mg/day, and the efficacy and safety of short-term everolimus therapy were examined. Finally, 82 TSC-AML patients were enrolled for analysis in this study. Of the 47 patients who underwent genetic testing, 22 patients (46.81%) had at least one detectable mutation in the *TSC1* or *TSC2* gene: 7 were *TSC1* gene mutations, 13 were *TSC2* gene mutations, and 2 were found in both *TSC1* and *TSC2*. Everolimus treatment had a statistically significant effect on the renal AML volume reduction during follow-up (*P* < 0.05), and the mean reduction rate of volume for all cases was 56.47 ± 23.32% over 12 weeks. However, 7 patients (7/25; 28.00%) experienced an increase in renal AML tumor volume within 12 weeks after discontinuation of the everolimus treatment. Although most patients (27/30, 90.00%) experienced some adverse events during the treatment period, all such events were mild, and no patients discontinued or needed dose reduction because of adverse events. Overall, in this study, the mutation rate of TSC-AML patients is much lower than other reports. Short-term everolimus treatment for TSC-AML is effective and safe, but the stability is much lower than long-term therapy.

## Introduction

Tuberous sclerosis complex (TSC) is a systemic autosomal dominant genetic disorder that affects approximately 1–2 million individuals worldwide^1^. It is usually caused by mutations in either the hamartin gene (*TSC1*) or tuberin gene (*TSC2*)^2,3^, leading to the growth of nonmalignant hamartomas in various organs throughout the body, including the kidney, brain, lung, skin, and heart^4–6^.

Statistical studies show that approximately 80% of patients with TSC develop renal angiomyolipoma (AML), which is rich in fat, muscle, and blood vessels^7^. Unlike sporadic renal AML, most TSC-related renal AML (TSC-AML) patients often develop multiple bilateral lesions and experience a significant tumor burden on the kidneys^8^. Furthermore, TSC-AML tend to be larger, grow faster and at higher risk of bleeding^9^. In addition, AML can increase in size over time and may cause hypertension, renal failure or life-threatening hemorrhage, which causes the largest proportion of adult deaths from the disease^10^.

The *TSC1* or *TSC2* gene mutation occurs in approximately 80% of patients with TSC^2,3^. The *TSC1* gene, located on chromosome 9q34, consists of 23 exons and codes for hamartin, a novel 130-kDa protein^3^. The *TSC2* gene, located on chromosome 16p13.3, contains 41 exons and codes for tuberin, a 200-kDa protein^2^. Hamartin and tuberin form a tumor suppressor complex that regulates the activity of rapamycin complex 1 (mTORC1), a critical regulator of cell growth and proliferation^11,12^. Mutations in *TSC1* or *TSC2* result in the loss of the hamartin/tuberin complex, which leads to constitutive activation of mTORC1. Increased mTORC1 signaling results in the production of hamartomatous lesions of TSC^12^.

Growing knowledge about the molecular relationship between TSC and mTOR has led to the investigation of mTORC1 inhibition as a treatment approach in TSC^6,13,14^. Moreover, the International Tuberous Sclerosis Complex Consensus Conference (ITSCCC) held in 2012 has recommended mTOR inhibitors as the first-line treatment for TSC-AML when enlarged to 3 cm or more^15^. Everolimus is a derivative of rapamycin that suppresses the enlargement of tumors and promotes their regression by inhibiting mTORC1^16^. Clinically, a randomized, double-blind, placebo-controlled EXIST-2 trial and extension studies have demonstrated the efficacy and manageable safety of everolimus for TSC-AML^17,18^.

In China, everolimus was included in national basic medical insurance, work injury insurance and the maternity insurance drug list in 2017. However, clinical information of TSC-AML patients in China and the effects of short-term everolimus therapy on TSC-AML are still limited. Thus, this study aimed to analyze the clinical characteristics and mutation spectra of patients with TSC-AML in Northwest China and to investigate the efficacy and safety of short-term everolimus therapy in patients with TSC-AML.

## Methods

This was an open single-center clinical study conducted at Xi Jing Hospital and was reviewed and approved by the Fourth Military Medical University ethics committee (ethics committee’s number: KY20151536-1). The study was performed in accordance with the principles of the Declaration of Helsinki and all local regulations. Written informed consent was obtained from all participants.

### Patient selection

From September 2015 to August 2018, a total of 167 patients with renal AML were screened at Xi Jing Hospital, 82 of whom were enrolled in this study. All 82 patients had been clinically (definitively or potentially) diagnosed with TSC per ITSCCC diagnostic criteria^15^. All patients underwent a systematic evaluation that covered 11 major criteria and 6 minor criteria of TSC before any interventions. Of these 82 patients, 47 patients (including 10 possible TSC-AML patients) were selected for the gene sequencing test, and 30 patients were recruited in the everolimus treatment trial.

Of the 30 patients who were selected for everolimus treatment, all had been definitively diagnosed with TSC, and patients with possible TSC were excluded. All of these patients were 18 years or older and had either multiple or bilateral AML that were 3-cm or larger at their largest diameter.

### Mutational analysis

Peripheral blood (10 mL) samples were collected from each patient and isolated from peripheral white blood cells. Then, DNA was extracted from peripheral blood leukocytes using standard methods. The mutational analysis of *TSC* genes was performed by next-generation sequencing (NGS), including the preparation of DNA libraries, the hybridization capture of target area and single-ended sequencing. Then, the data were analyzed, and all variation results were validated by the PCR-SSCP method (all genetic tests were performed by KANGSO Medical Institute, Beijing China). Finally, all of the mutations were compared with the Tuberous Sclerosis Database (www.lovd.nl/TSC1; www.lovd.nl/TSC2).

### Everolimus treatment

Everolimus was administered orally at a dose of 10 mg/day, and the levels of everolimus in blood were measured at 2 weeks to ensure blood everolimus concentrations were between 5 and 15 ng/ml. All patients received everolimus for 12 weeks, while 25 patients were followed for an additional 12 weeks after the therapy was stopped.

The primary endpoint of this study was the efficacy of short-term everolimus therapy, which was assessed according to the reduction in the volume of renal AML. The volume of renal AML was evaluated using kidney magnetic resonance imaging or CT scanning, and all scans were assessed by central radiological review. The volume of renal AML before initiation of everolimus treatment was defined as “baseline”. Follow-up visits were performed at week 4 and 12 after the start of everolimus treatment and at week 12 after the treatment stopped. Treatment efficacy was defined as the volume reduction rate compared to the baseline, which was calculated with the formula: (V_Pre-Therapy_ − V_Post-Therapy_)/V_Pre-Therapy_ × 100%. (V_Pre-Therapy_ represents pre-everolimus therapy volume; V_Post-Therapy_ represents post-everolimus therapy volume).

The secondary endpoint of this study was the frequency and severity of adverse events (AE). AEs were evaluated using a structured questionnaire covering the side effects reported during EXIST 2 or extension studies and were coded using the Medical Dictionary for Regulatory Activities (MedDRA) preferred term^17,19^. Furthermore, adverse events were continuously monitored in every follow-up visit and graded I-V according to the Common Terminology Criteria for Adverse Events (CTCAE) of the National Cancer Institute version 4.03 via investigator assessment^20^. To assess safety, a complete blood count, urinalysis and the levels of electrolytes (blood urea nitrogen, creatinine, glucose, hepatic enzymes, bilirubin, and serum lipids) were also tested in every patient. All laboratory tests were performed by the Department of Laboratory, Xi Jing Hospital, the Fourth Military Medical University.

### Statistical analysis

Clinical characteristics were collected from all patients screened for TSC-AML during the experiment period, and the results were presented as a frequency distribution (absolute frequencies and valid percentages; n, %). The mutation spectra were the statistical result of the 47 patients who agreed to participate in the genetic testing, and the results were expressed in terms of frequency distribution. Efficacy and safety analyses were performed on all patients who received at least 12 weeks of everolimus treatment and had at least one follow-up assessment; missing data were not considered in the analyses. Descriptive analyses of patient characteristics were conducted, including central tendency and dispersion (mean ± standard deviation [SD]), or median and frequency distribution. The outcomes were analyzed using a *t*-test or χ^2^ test, and a *P*-value < 0.05 was considered statistically significant. All statistical analyses were performed with the Statistical Package for the Social Sciences (SPSS, SPSS Inc, Chicago, USA) version 17.0.

## Results

### Clinical characteristics of patients with TSC-AML

A total of 82 Chinese patients with TSC-AML, including 65 definitively diagnosed patients and 17 patients with possible TSC-AML were enrolled in this study since 2015. The patients were 35 ± 12 years old (range 18–68 years), with a median age of 36 years. Among all patients, 28 were males (34.15%), and 54 were females (65.85%) (Table 1).

**Table 1:** Clinical characteristics of TSC-AML patients.

	All (N = 82)	TSC2 mutation (n = 15)	Non-TSC2 mutation(n = 32)	*P* value
**Age (years)**				
Mean ± SD	35 ± 12	35 ± 9	38 ± 12	0.12
Median age	36	34	42	
**Sex**				
Male	28 (34.15)	6	10	0.56
Female	54 (65.85)	9	22	
**Major features**				
Hypomelanotic macules (≥3)	38 (46.34)	11 (73.33)	10 (31.25)	0.007
Angiofibromas (≥3) or fibrous cephalic plaque	73 (89.02)	14 (93.33)	27 (84.38)	0.39
Ungual fibromas (≥2)	33 (40.24)	8 (53.33)	10 (31.25)	0.15
Shagreen patch	21 (25.61)	6 (40.00)	6 (15.63)	0.12
Multiple retinal hamartomas	3 (3.66)	1 (6.67)	—	—
Cortical dysplasias	18 (21.95)	4 (26.67)	6 (18.75)	0.54
SEN	26 (31.71)	6 (40.00)	7 (21.89)	0.20
SEGA	2 (2.44)	1 (6.67)	—	—
Cardiac rhabdomyoma	—	—	—	—
LAM (Women)	19 (35.19)	5 (55.55)	6 (27.27)	0.14
AML (≥2)	82 (100.00)			
Diameter_max < _10 cm	44 (53.66)	5 (33.33)	21 (65.63)	0.038
Diameter_max_ ≥ 10 cm	38 (46.34)	10 (66.67)	11 (34.38)	
Diameter_max_ (mean ± SD, cm) (range, cm)	11.23 ± 6.47 (3.31–28.34)	16.37 ± 9.61 (4.42–22.54)	9.14 ± 6.84 (3.31–20.33)	0.042
**Minor features**				
“Confetti” skin lesions	42 (51.22)	9 (60.00)	12 (37.50)	0.15
Dental enamel pits (>3)	23 (28.04)	6 (40.00)	8 (25.00)	0.29
Intraoral fibromas (≥2)	19 (23.17)	5 (33.33)	6 (18.75)	0.30
Retinal achromic patch	5 (6.10)	3 (20.00)	1 (3.13)	0.05
Multiple renal cysts	3 (3.66)	1 (6.67)	1 (3.13)	0.57
Nonrenal hamartomas	1 (1.22)	1 (6.67)	—	—

TSC, tuberous sclerosis complex; AML, angiomyolipoma; SD, standard deviation; SEN, subependymal nodule; LAM, lymphangioleiomyomatosis; SEGA, subependymal giant cell astrocytoma; Non-TSC2 mutations: TSC1 mutations and nonmutations. The outcomes of frequency distribution were analyzed using χ^2^ test and the central tendency and dispersion data (mean ± standard deviation [SD]) was analyzed using *t*-test. *P*-value refers to the comparison between *TSC-2* mutation group and non-*TSC2* mutation group, and *P* < 0.05 was considered statistically significant.

All patients underwent a comprehensive assessment before any interventions, including both physical and radiographic aspects. In addition, all clinical features were collected using a standardized clinical instrument that covers 11 major criteria and 6 minor criteria of TSC^15^. As shown in Table 1, all patients had at least two AMLs, with the largest diameter (≥3 cm). Among them, there were 44 (53.66%) patients with AMLs with the largest diameter (<10 cm), 38 (46.34%) patients with AMLs with the largest diameter (≥10 cm), and the average AMLs with the largest diameter of patients was 11.23 ± 6.47 cm (range 3.31–28.34 cm). Of these patients, all except 9 patients (89.02%) had angiofibromas or fibrous cephalic plaques, 38 patients (46.34%) exhibited hypomelanotic macules, and 33 patients (40.24%) had at least two ungual fibromas. Furthermore, 21 patients (25.61%) presented with shagreen patches, 3 patients (3.66%) had multiple retinal hamartomas, and 18 patients (21.95%) exhibited cortical dysplasias. In addition, subependymal nodules were present in 26 patients (31.71%), and subependymal giant cell astrocytoma was found in 2 patients (2.44%). In the 54 female patients, 19 patients (35.19%) were identified as having lymphangioleiomyomatosis (Table 1).

In minor features aspects, “confetti” skin lesions were the most common, affecting 42 patients (51.22%), followed by dental enamel pits (23 patients; 28.04%), intraoral fibromas (19 patients; 23.17%) and retinal achromatic patches (5 patients; 6.10%). In addition, 3 patients (3.66%) presented with multiple renal cysts, and 1 patient (1.22%) had nonrenal hamartomas (Table 1).

### Mutation spectra and novel sequence variants

Out of 47 Chinese TSC-AML cases, *TSC* gene mutations were found in 22 patients (46.81%). Among 22 *TSC* gene mutations, 7 (14.89%) were *TSC1* gene mutations, 13 (27.66%) were *TSC2* gene mutations and 2 (4.26%) were both *TSC1* and *TSC2* gene mutations. Of these 22 patients, a total of 26 mutations were detected, and 3 patients had more than 1 type of mutation. Of all mutations, a total of 21 different kinds of mutations were identified, 5 in *TSC1* and 16 in *TSC2* (Table 2).

**Table 2:** Mutations in *TSC1* and *TSC2* of TSC-AML patients.

Case	Age	Sex	Genotype and location	Nucleotide change	Amino acid change	Mutaion type	Status
1	68	M	*TSC1*: Exon 15	c.1921C > T	p.Pro641Ser	Missense	Novel
2	53	F	*TSC2*: Intron 1	c.1-34 G > C	—	Splicing	Novel
3	34	M	*TSC1*: Exon 10	c.965 T > C	p.Met322Thr	Missense	Reported
4	46	M	*TSC2*: Exon 21	c.2252 G > A	p.Arg751Gln	Missense	Reported
5	27	M	*TSC2*: Intron 1 *TSC2*: Exon 19 *TSC2*: Exon 41	c.1-35 G > A; c.2032 G > A; c.5227 C > T	– p.Ala678Thr; p.Arg1743Trp	Splicing Missense Missense	Novel Reported Reported
6	23	F	*TSC2*: Exon 28	c.3179 G > A	p.Trp1060Ter	Nonsense	Novel
7	35	F	*TSC2*: Exon 17	c.1830delC	p.Arg611GlyfsTer87	Frameshift	Novel
8	34	F	*TSC2*: Exon 9	c.826_827del	p.Met276ValfsTer61	Frameshift	Reported
9	23	M	*TSC2*: Exon 20	c.2106_2107insT	p.Trp703LeufsTer13	Frameshift	Novel
10	29	F	*TSC2*: Exon 1–42	—	—	Large Deletion	Novel
11	62	F	*TSC1*: Exon 10	c.965 T > C	p.Met322Thr	Missense	Reported
12	39	M	*TSC1*: Exon 10; *TSC2*: Exon 18	c.965 T > C; c.1911C > T	p.Met322Thr; Thr; p.Val637Val;	Missense Missense	Reported Novel
13	49	F	*TSC1*: Exon 5;	c.250 G > A	p.Ala84Thr	Missense	Reported
14	41	F	*TSC1*: Exon 15	c.1631G > A	p.Gly544Glu	Missense	Reported
15	31	F	*TSC2*: Exon 16	c.1659_1660insTCGG	p.Ser554fs	Frameshift	Novel
16	29	F	*TSC2*: Exon19	c.2032 G > A	p.Ala678Thr	Missense	Reported
17	23	F	*TSC1*: Exon 10	c.965 T > C	p.Met322Thr	Missense	Reported
18	43	M	*TSC2*: Exon 19	c.2083 C > T	p.Gln695Ter	Nonsense	Reported
19	46	M	*TSC1*: Exon 14 *TSC2*: Exon 33	c.1335 A > G c.3986 G > A	p.Glu445Glu; p.Arg1329His	Missense Missense	Reported Reported
20	31	F	*TSC2*: Exon 41	c.5227_5244del	p.1743_1748del	Deletion	Novel
21	43	F	*TSC1*: Exon 5	c.250 G > A	p.Ala84Thr	Missense	Reported
22	39	F	*TSC2*: Exon 1–6	Exon 1-6del	—	Large Deletion	Novel

M, Male; F, Femal.

As shown in Table 2, the mutations in *TSC1* and *TSC2* can be distributed over the entire *TSC1* and *TSC2* gene regions; most of them were in exons (24/26; 92.31%), and only 2 of them were in introns (7.69%). Among *TSC1* mutations, c.965 T > C was the most common mutation, which can be found in 4 cases. The c.965 T > C mutation is a type of missense mutation located in exon 10 of the *TSC1* gene, and this mutation results in a p.Met322Thr amino acid change. Among *TSC2* mutations, c.2032 G > A was the most common mutation and was involved in 2 cases. c.2032 G > A is also a type of missense mutation located in exon 19 of the *TSC2* gene, and this mutation results in a p.Ala678Thr amino acid change. Among all mutation types, missense mutations were the most common, accounting for 57.69% (15/26), followed by frameshift mutations (4/26), deletions (3/26, including 2 large deletions), nonsense mutations (2/26) and splicing mutations (2/26) (Table 2). In addition, eleven novel nucleotide alterations that have not been registered in the LOVD database were identified in the present study, 10 of which are in the *TSC2* gene: (c.1–34 G > C, c.1–35 G > A, c.3179 G > A, c.1830delC, c.2106_2107insT, c.1911C > T, c.1659_1660insTCGG, c.5227_5244del and exon 1-6del) and 1 in the *TSC1* gene (c.1921C > T) (Table 2).

**Figure 1:**
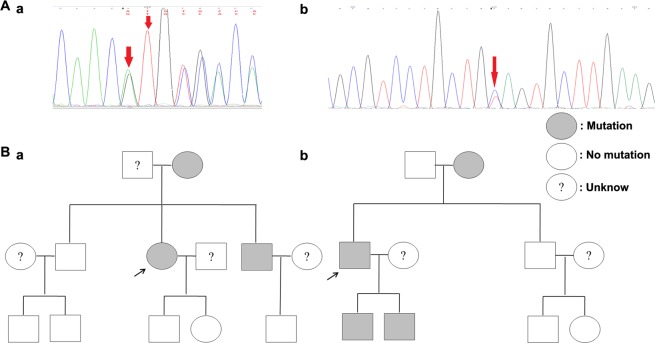
Sequencing results and mutant pedigree map of familial mutations. (**A**) Sequencing results of two familial mutations. The red arrow indicates the point of mutation. (a) Sequencing results of mutation c.826_827del; (b) Sequencing results of mutation c.2083 C > T. (**B**) Mutant pedigree map of familial mutations. The arrow refers to the proband; square represents the male; circle represents the female; (**a**) Mutant pedigree map of mutation c.826_827del; **b:** Mutant pedigree map of mutation c.2083 C > T.

Among these 22 patients with *TSC1* or *TSC2* mutations, 7 of these patients’ families also underwent genetic tests and found that two of the mutations were familial, including c.826-827del, located in exon 9 of the *TSC2* gene and c.2083 C > T, located in exon19 of the *TSC2* gene (Fig. 1). The c.826-827del mutation was a kind of frameshift mutation, and this mutation results in p.Met276ValfsTer61 amino acid change. A total of 9 family members underwent *TSC* genetic testing, of which 3 exhibited the same mutation, namely, the patient, the mother and the younger brother. The c.2083 C > T mutation was a type of nonsense mutation resulting in a p.Gln695Ter amino acid change. For this case, the *TSC* genetic tests were performed in 8 family members, and 4 of them carried the same mutation, namely, the patient, the mother and two sons (Fig. 1).

### Comparison between *TSC2* and non-*TSC2* mutation populations

Within the group of patients who underwent a gene sequencing test, we compared the clinical features of patients with *TSC2* mutations with patient populations with non-*TSC2* mutations (*TSC1* mutation or no mutation). As shown in Table 1, patients with *TSC2* mutations had a significantly larger average AML largest diameter compared with patient populations with non-*TSC2* mutations (16.37 ± 9.61 cm vs 9.14 ± 6.84 cm, *P* = 0.042). Ten patients (66.67%) with *TSC2* mutations had a AML largest diameter of ≥10 cm, compared to only 11 patients (34.38%) in the non-*TSC2 *mutation group (*P* = 0.038). In addition, hypomelanotic maculesoccurred more frequently in the *TSC2* mutation group (*P* = 0.007). Although the difference was not statistically significant between these two groups for the other clinical features, the *TSC2* mutation group was younger and had a higher frequency of other major or minor features (Table 1).

### Treatment efficacy

A total of 30 patients who had been definitively diagnosed with TSC-AML were enrolled in the everolimus treatment study. Of the 30 patients, the median age was 35 (range 18–52) years, 17 were ≤ 35 years old (56.67%) and 13 were >35 years old (43.33%). Overall, the majority of patients (63.33%; 19/30) were female, and 11 patients (36.67%) were male. Twelve patients (40.00%) had a primitive volume (>200 cm^3^) of the largest renal AML at baseline. Genetic testing was performed in 19 patients, 5 with mutations to *TSC1* or *TSC2* (1 with mutations in *TSC1*, 4 with mutations in *TSC2*) and 14 with no mutations (Table 3).

**Table 3:** Patient demographics and disease characteristics in treatment group.

Characteristics	All (N = 30)	%
Median age (range), years	35 (18–52)	
**Age**		
**≤**35 years	17	56.67
**>**35 years	13	43.33
**Sex**		
Male	11	36.67
Female	19	63.33
**Primitive volume of the largest renal AML**		
**≤**200 cm^3^	18	60.00
>200 cm^3^	12	40.00
**TSC1 or TSC2 mutation**		
With mutation	5	16.67
None mutation	14	46.67
Unknown	11	36.67

All of the patients were given everolimus orally at doses of 10 mg per day. The duration of administration was 12 weeks. After 4 and 12 weeks of therapy, the mean renal AML volume decreased 36.51 ± 23.94% (range: 4.23%-84.47%; *P* < 0.05 for the change from the baseline value) and 56.47 ± 23.32% (range: 4.88%-97.36%; *P* < 0.05 for the change from the baseline value), respectively. In addition, we found that male patients or those with primitive volume (<200 cm^3^) experienced a better efficacy of treatment when they took everolimus for 12 weeks. However, there was no significant difference in the efficacy of treatment with respect to age and mutation type (mutation group vs no mutation group) (Table 4).

**Table 4:** Change in volume of renal AML following everolimus treatment.

Mean ± SD/Range	Classification	4 weeks (%)	*P* value	12 weeks (%)	*P* value
All	N = 30	36.51 ± 23.94 (4.23–84.87)	1.57E-11^#^*	56.47 ± 23.32 (4.88–97.36)	3.25E-19^#^*
Age	≤35 years (n = 17)	32.87 ± 24.85 (4.23–84.49)	0.25	51.80 ± 26.12 (4.88–97.36)	0.26
	>35 years (n = 13)	41.28 ± 22.76 (8.10–84.87)		62.58 ± 18.24 (37.46–90.71)	
Sex	Male (n = 11)	38.38 ± 119.58 (8.34–74.99)	0.75	68.55 ± 11.68 (54.48–90.71)	0.03*
	Female (n = 19)	35.43 ± 26.59 (4.23–84.87)		49.47 ± 25.67 (4.88–97.36)	
Primitive Volume	<200 cm^3^ (n = 18)	39.53 ± 22.69 (8.10–84.87)	0.41	64.70 ± 16.79 (34.72–90.71)	0.02*
	≥200 cm^3^ (n = 12)	31.99 ± 26.04 (4.23–84.49)		44.12 ± 26.84 (4.88–97.36)	
Mutation	With Mutation (n = 5)	28.95 ± 19.77 (6.93–51.04)	0.51	44.13 ± 25.03 (15.92–77.64)	0.43
	No Mutation (n = 14)	37.05 ± 23.79 (7.87–84.87)		54.25 ± 23.88 (4.88–90.05)	

^#^Means comparison with baseline.

*Means *P* < 0.05 and the difference is statistically significant.

**Figure 2:**
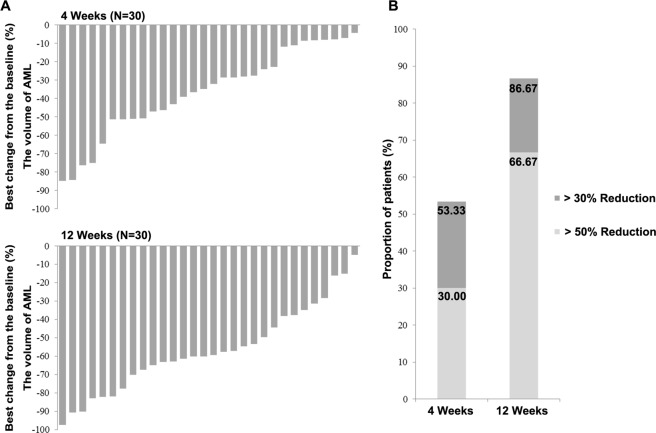
Magnitude of treatment effect. (**A**) Best percentage changes of largest renal AML volume from baseline. **(B**) The propotion of patients with tumor volume reduction >30% and >50%.

The reduction rate of volume from the baseline in each case is shown in Fig. 2A. All except 1 patient responded to everolimus (96.67%; 29/30), with a statistically significant volume reduction (>10% compared with baseline) after 12 weeks of treatment, and the largest rate of volume reduction from the baseline was 97.36% (882.47 cm^3^ to 23.26 cm^3^) after 12 weeks of therapy. However, the renal volume of the nonresponder only experienced a 4.88% (773.31 cm^3^ to 735.61 cm^3^) reduction after 12 weeks of treatment (Fig. 2A). The proportion of patients who achieved ≥30% reduction from baseline in renal AML volume increased from 53.33% (16/30) after 4 weeks of treatment to 86.67% (26/30) at week 12, and the proportion of patients with ≥50% reduction increased from 30.00% (9/30) after 4 weeks of treatment to 66.67% (20/30) after 12 weeks (Fig. 2B).

**Figure 3:**
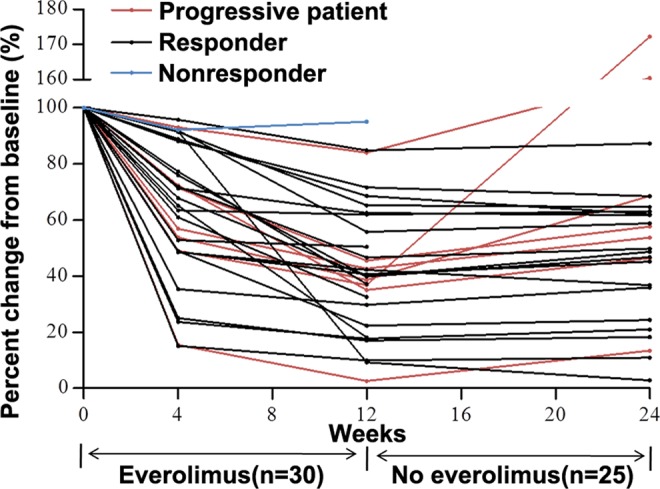
Change of the volume along time with everolimus treatment. Red represents progressive patient, black represents responder and blue represents nonresponder.

As shown in Fig. 3, in most patients, the AML volume decreased rapidly within 4 weeks after treatment initiation, but the rate of decrease significantly slowed down for the next 8 weeks. However, 7 of these patients (7/25; 28.00%) experienced an increase in renal AML volume within 12 weeks after discontinuation of the everolimus treatment (an increase larger than 10% compared with baseline). In addition, among these 7 progression patients, 2 patients experienced renal AML lesions increased over baseline levels, and the volume had increased to 160.41% (781.68 cm^3^ to 1253.93 cm^3^) and 172.19% (80.09 cm^3^ to 137.93 cm^3^) of the baseline, respectively. Despite this progression, all except one nonresponder and two progressive patients exhibited at least a 10% reduction in volume compared with baseline by the cut-off day.

### Adverse events

The AEs were monitored throughout the treatment period and and no patients took other special drugs during this time. As shown in Table 5, a wide variety of everolimus treatment-related AEs occurred, and they were consistent with the known safety profile of everolimus^17,18^, which most commonly included stomatitis, headache, acne, cough and menstrual disorders.

**Table 5:** Treatment-related adverse events.

Event	All grades No.(%)	Grade 1	Grade 2	Grade 3	Grade 4
Stomatitis	27 (90.00)	26	1	0	0
Headache	11 (36.67)	11	0	0	0
Upper respiratory tract infection	5 (16.67)	4	1	0	0
Urinary tract infection	0	0	0	0	0
Nausea	4 (13.33)	4	0	0	0
Vomit	5 (16.67)	5	0	0	0
Acne-like skin lesions	8 (26.67)	8	0	0	0
Fatigue	4 (13.33)	4	0	0	0
Cough	8 (26.67)	8	0	0	0
Hypercholesterolemia	4 (13.33)	4	0	0	0
Bleeding	0	0	0	0	0
Cytopenia	4 (13.33)	3	1	0	0
*Menstrual disorders	4 (23.53)	0	4	0	0

^*^Percentage statistics only record the total number of non-menopausal female patients (n = 17).

In our study, all AEs noted were very mild, with no grade 3 or 4 AEs detected, and no patients were hospitalized with serious AEs. Overall, 27 patients (90.00%) experienced grade 1 or 2 AEs during the study period, and they all could be managed successfully through symptomatic treatment; no one needed dose reduction or temporary interruption of treatment. Notably, stomatitis was the most common AE, and it was reported in 27 patients, of whom 26 were grade 1 and 1 was grade 2. By corresponding oral care, all cases were improved in 4 weeks. Headache was reported in 11 patients (36.67%) and was the second most common AE. Upper respiratory infections in 4 cases (13.33%) of grade 1 and 1 case (3.33%) of grade 2 were detected, but no urinary tract infection was reported. In addition, 9 patients (30.00%) developed digestive system AEs, including nausea (4/30, 13.33%) and vomiting (5/30, 16.67%). Regarding severity, the most common grade 2 AEs were menstrual disorders. In nonmenopausal female patients (17patients), a total of 4 (23.53%) patients experienced menstrual disorders, all of which resolved in 8 weeks without any hormone treatment.

During the treatment period, everolimus treatment-related laboratory tests were also monitored, including blood, urine, liver and kidney functions. No other abnormalities were observed, except for 4 cases (13.33%) of hyperlipidemia (grade 1) and cytopenia (3 cases of grade 1 and 1 case of grade 2).

## Discussion

TSC was initially described in 1862 by von Recklinghausen. It is an extremely variable disease that can affect virtually any organ in the body^1^. In the late nineteenth century, it was identified by simple clinical observations, such as epilepsy, cognitive retardation, and facial angiofibroma. Then, with other manifestations discovered and its pathogenesis revealed, the diagnostic criteria for TSC have also improved over time^21^. In 2012, the International Tuberous Sclerosis Complex Consensus Group holds the ITSCCC and update TSC diagnostic criteria^15^. The new diagnostic criteria includes new genetic testing results, reduced diagnostic classes from three (possible, probable, and definite) to two (possible, definite) and some minor changes to specific criteria^15^. However, the clinical features of TSC continue to be a principal means of diagnosis.

The clinical manifestations of TSC are quite diverse and distinctive, including lesions of the brain, skin, heart, lungs, retina and kidneys. All these manifestations are divided into 11 major features and 6 minor features^15^. In this study, we reported the clinical features of 82 patients with TSC-AML in northwestern China. In addition to renal AML, angiofibromas (≥3) or fibrous cephalic plaques were the most common major features in TSC-AML patients and were identified in 89.02% (73/82) of patients. In minor features aspects, confetti skin lesions were the most common in all patients. Similar results were reported by Yi Cai^22^.

In addition to diagnosis by clinical diagnostic criteria, the genetic analysis was also an independent diagnostic criterion for TSC^15^. As the molecular biology and genetics that underly the pathophysiologic process of TSC were elucidated in the late years, comprehensive and reliable screens for TSC gene mutations are well established, and more pathogenic mutations have been identified^21^. *TSC1* and *TSC2* genes are both pathogenic mutations of TSC, and approximately 80% of patients with a clinical diagnosis of TSC have exhibited gene mutations^2,3^.*TSC2* mutations are more common than *TSC1* mutations, and *TSC2* has been shown to be mutated in approximately 70% of TSC patients, whereas *TSC1* mutations can only be found in 20% of TSC cases^2,3^. Furthermore, *TSC2* mutations are associated with a more severe phenotype and a higher frequency and larger renal AML size compared with non-*TSC2* mutations^22–24^. Our study showed similar results: patients with *TSC2* mutations had larger AMLs and a higher frequency of other clinical features compared with patient populations with non-*TSC2* mutations.

In this experiment, we examined mutations of the*TSC1* and *TSC2* genes in 47 TSC-AML patients. Of these patients, gene mutations were found in 22 patients: 7 were *TSC1* gene mutations, 13 were *TSC2* gene mutations and 2 were *TSC1* and *TSC2* gene mutations. In comparing our study with previous reports, the mutation rates are lower than those of other regions (other parts of China)^22,23,25–29^. The low detection mutation rate found in this study could be due to: (1) clinical possibly TSC patients may had lower gene mutation rates (there were 10 clinical possibly patients also underwent genetic testing); (2) mutations in intronic and promoter regions, which might disrupt gene expression, and be missed by most mutation screening methods; (3) difficulty of detecting mutations by any conventional method in patients with diagnostic features of tuberous sclerosis and low rate of mosaicism for either TSC1 or TSC2 mutations^30,31^; (4) additional causative loci that could account for a few patients with tuberous sclerosis and we could not detect it using conventional screening methods^32,33^; (5) the specificity of the population in Northwest China. However, due to the limited sample size, this conclusion needs to be deeply verified. For all these reasons, it is still difficult to assert that the genetic mutation rate of TSC patients in Northwest China is lower than other regions. Therefore, more accurate researches are needed to further verify the findings in this study.

NGS is a powerful technology that allows for a more accurate, more depth and less time-consuming genetic analysis that was never before possible^32^. To date, 928 unique DNA variants of *TSC1* (update to September 03, 2018) and 2689 unique DNA variants of *TSC2* (update to September 24, 2018) have been reported and listed on the TSC Variation Database site (www.lovd.nl/TSC1; www.lovd.nl/TSC2). In this study, a total of 21 different types of mutations were identified, 11 (52.38%) of which were newly discovered. Such a high rate of novel mutations may be due to racial specificity, which may also be another reason for the low mutation rate. Similarly, the literature shows that the TSC mutation spectra and rates vary from country to country^21,22,33–37^. A previous study showed that alterations in *TSC1* and *TSC2* can be distributed over the entire *TSC1* and *TSC2* gene regions. Our study showed similar results. Of these 21 mutations, 5 were located in *TSC1*, and 16 were in *TSC2*. These mutations were distributed in the exon or intron regions of the *TSC1* and *TSC2* genes, but the most common regions in the *TSC1* and *TSC2* genes were exon 10 and exon 19, respectively. Moreover, in *TSC2* mutations, there were 2 nucleotide alterations that were identified as familial mutations; three of the 9 family members (c.826_827del) and four of 8 family members (c.2083 C > T) showed the same mutation. Although there are many advantages of NGS, and it can definite diagnosis TSC, the high cost of sequencing makes it impossible for many poor areas to diagnose all patients suspected of TSC by gene sequencing^32^.

In patients with TSC, renal problems (renal failure or tumoral complications, retroperitoneal hemorrhage, and metastases of renal cell carcinoma) is the second leading cause of death or disability, second only to severe intellectual disability^38,39^. The cause of death in TSC-AML patients is associated with progressive increases in size, which in turn lead to renal failure or spontaneous hemorrhage^40,41^. Thus, current guidelines recommend that the key goal of treatment for TSC-AML is to prevent renal AML progressive enlargement, thereby ameliorating the risk of future bleeding, impaired renal function and mortality^15^. Hence, the 2012 ITSCCC recommended mTOR inhibitors as the first-line therapy for asymptomatic renal AML measuring larger than 3 cm in diameter^15^. Two mTOR inhibitors, sirolimus and everolimus, have been evaluated for their efficacy and safety in the treatment of various manifestations associated with TSC^42,43^. Everolimus is an oral mTOR inhibitor derived from sirolimus, which had been approved by FDA in the treatment of patients with TSC-AML and adult patients with TSC-subependymal giant cell astrocytoma^44^, and the improved pharmacokinetic profile of everolimus over sirolimus makes it an attractive, noninvasive option for patients. Its efficacy and safety in the treatment of TSC-AML had been investigated in EXIST-2 and extension studies recently; the clinical trial found 54.93% of patients (39/71) showed 50% shrinkage of the tumors after they were administered everolimus for 6 months, and a pronounced benefit was demonstrated with continued use of everolimus^17,18^. In the present study, we tested the efficacy and safety of short-term everolimus therapy for TSC-AML. The results revealed that the volume of AML decreased 50% or more in 63.33% (19/30) of patients after 12 weeks of everolimus therapy, which showed a similar tumor size-reducing effect with prior studies^45–47^. Furthermore, the reduction rate of tumor size will gradually slow with the extension of treatment time, and these results are consistent with data reported by the EXIST-2 and extension trials^17,18^. However, our research shows that 7 patients (7/25; 28.00%) who were primarily sensitive to everolimus treatment, experienced an increase in renal AML tumor volume after the treatment stopped. Although similar results have been reported in other studies^48–50^, the AML progression rate was much higher in this study than in other long-term everolimus treatment studies. This may be due to a number of reasons, but the main reason is that the treatment time is short and possibly related to the sensitivity of the Chinese race to everolimus treatment. Overall, compared with long-term (≥6 months) everolimus treatment, the reduction rate of renal AML volume in short-term use of everolimus was similar, but with a higher risk of developing renal AML progression after discontinuation. This finding potentially implies that long-term use of everolimus in the treatment of TSC-AML results in more stable treatment efficacy than shorter-term use.

In addition to the treatment efficacy of everolimus for TSC-AML, a wide variety of everolimus treatment-related adverse events have also been reported^17,18,51–53^. Reported adverse events of everolimus include stomatitis, headache, infections, vomiting, hypercholesterolaemia, and irregular menstruation^17,18^. In the EXIST-2 extension study, nasopharyngitis (43%), stomatitis (43%) and headache (30%) were the most common adverse events with everolimus therapy^18^. In previous observations, most of the adverse events of everolimus treatment were grade 1–2, and few patients discontinued treatment for adverse events^18^. Our study showed similar results; although most patients presented with side effects, no patients discontinued or needed dose reduction because of adverse events, all of which were grade 1 or 2 and reversible. Menstrual disorders were also reported in this study, which occurred at a high rate and was the most common grade 2 adverse event in the nonmenopausal female group (4/17; 23.52%). Although the cause of menstrual disorders is still not very clear, some researchers think it may be caused by mTOR inhibitors disturbed hormone levels^53–55^. Because many TSC-AML patients are young, reduction of gonadal function should be explained to patients before the initiation of everolimus treatment. Overall, treatment with everolimus was safe during the observation period, and all adverse events were mild and manageable in this study.

Several limitations should be emphasized in the present study. Because of the relatively small sample size and because only descriptive statistics were available from a single center, a large multicenter study is needed in the future. A further limitation was the short observation time. In the present study, we only observed 12 weeks after the treatment stopped, and a comprehensive assessment of the long-term effects of short-term use of everolimus in the treatment of TSC-AML could not be performed.

## Conclusions

In summary, this open single-center clinical study explored the clinical features and gene mutation information of TSC-AML patients in Northwest China and observed the efficacy and safety of short-term use of everolimus in patients with TSC-AML. Overall, the *TSC* gene mutation rate of TSC-AML patients in this study is much lower than in other reports. The short-term everolimus treatment for TSC-AML is effective and safe, but the stability is much lower than long-term therapy.
